# Long-Term Survival after Repeat Resection and Multimodal Therapy for Primary Cardiac Angiosarcoma: A Case Report

**DOI:** 10.70352/scrj.cr.25-0785

**Published:** 2026-07-01

**Authors:** Keiichi Ishida, Shoichi Takahashi

**Affiliations:** Department of Cardiovascular Surgery, Hoshi General Hospital, Koriyama, Fukushima, Japan

**Keywords:** primary cardiac angiosarcoma, right atrial tumor, complete resection, repeat resection, multimodal therapy

## Abstract

**INTRODUCTION:**

Primary cardiac angiosarcoma (PCA) is an exceptionally rare and aggressive malignant tumor characterized by a poor prognosis. Although complete surgical resection is the most effective treatment for survival, recurrence and metastasis occur frequently even after radical excision.

**CASE PRESENTATION:**

We report a rare case of PCA in a 40-year-old woman in whom long-term survival of 68 months was achieved through aggressive multimodal therapy. She underwent urgent complete resection of a right atrial tumor, followed by preoperative chemotherapy and repeat resection with tricuspid valve replacement for recurrent disease and pulmonary metastasis. Postoperative adjuvant chemotherapy resulted in disease-free survival of 41 months. The subsequent hepatic and pulmonary metastases were managed with chemotherapy and radiotherapy before the patient succumbed to multiple organ failure.

**CONCLUSIONS:**

Individualized, aggressive, and multimodal treatment—including repeat surgical resection and sustained systemic therapy—can achieve durable disease control and prolonged survival in selected patients with PCA, a malignancy otherwise associated with extremely poor outcomes.

## Abbreviations

IVC
: inferior vena cava
PCA
: primary cardiac angiosarcoma
TTE
: transthoracic echocardiography
TVR
: tricuspid valve replacement

## INTRODUCTION

PCA is a rare and highly aggressive malignant tumor, with a predilection for the right atrium in middle-aged men (mean age: 39–48.9 years).^1)^ Its prognosis is extremely poor, with a median survival ranging from 6 to 13 months; moreover, up to 80% of patients experience metastatic disease at the time of diagnosis.^1–3)^ Multimodal treatment, which combines surgery, chemotherapy, and radiotherapy, is the cornerstone of management. Notably, complete (R0) resection significantly improves outcomes, extending median survival to approximately 17 months.^1)^ Although combined chemoradiotherapy may further prolong survival to approximately 27 months,^4)^ most patients ultimately succumb to early recurrence or metastasis despite aggressive therapy.^1)^

Herein, we present a rare case of PCA in which a long-term survival of 68 months was achieved following complete resection, repeat recurrence surgery, and adjuvant chemotherapy.

## CASE PRESENTATION

**Fig. 1:**
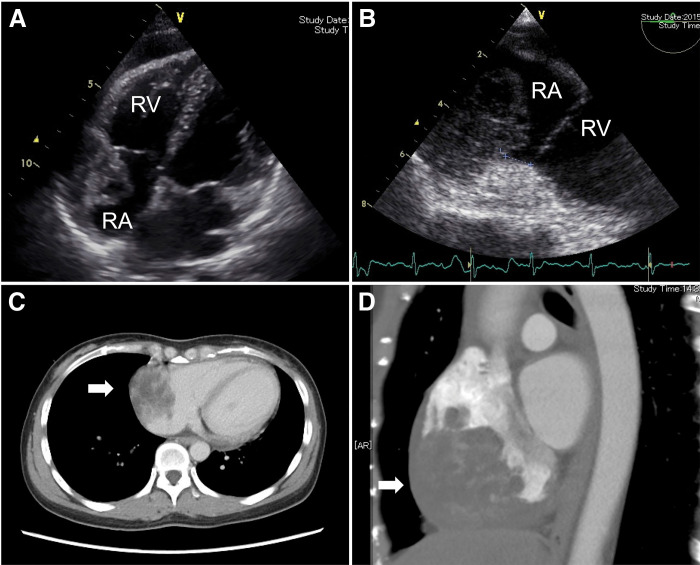
Preoperative imaging findings. (**A**) TTE demonstrating a large mass occupying the right atrium, accompanied by pericardial effusion. (**B**) Transesophageal echocardiography revealing a 55 × 41-mm non-pedunculated mass with heterogeneous internal echogenicity, arising from the anterior and superior right atrial wall without tricuspid valve involvement. (**C**, **D**) Contrast-enhanced CT showing an irregularly contoured and heterogeneously enhanced mass measuring 54 mm in maximal diameter along the free wall of the right atrium (arrows). RA, right atrium; RV, right ventricle; TTE, transthoracic echocardiography

A 40-year-old woman presented with easy fatigability for approximately 2 weeks and was referred to our hospital due to liver dysfunction and pericardial effusion identified on TTE. Her medical history included an endometrial polypectomy. Her family history was insignificant. Upon admission, her vital signs were stable, and physical examination yielded no notable findings. Blood tests indicated elevated tumor markers (CA-125: 89.4 U/mL). TTE revealed a mass in the right atrium, accompanied by mild tricuspid regurgitation and pericardial effusion (**Fig. 1A**).

Transesophageal echocardiography revealed a non-pedicled mass measuring 55 × 41 mm, exhibiting mottled, hypoechoic areas within the upper and anterior walls of the right atrium, without involvement of the tricuspid valve (**Fig. 1B**). Contrast-enhanced CT demonstrated an irregular right atrial mass with heterogeneous enhancement measuring 54 mm along the major axis (**Fig. 1C** and **1D**). On MRI, the mass showed intermediate signal intensity with scattered high-signal intensity, high-signal intensity with scattered low-signal intensity, and scattered high-signal intensity on T1-weighted, T2-weighted, and diffusion-weighted sequences, respectively. Neither CT nor MRI revealed additional mass lesions in other organs. These findings were indicative of malignancy, and early histopathological confirmation was considered essential for appropriate oncologic management. The patient showed progressive deterioration with worsening fatigue, liver dysfunction, and pericardial effusion, indicating right heart failure. Urgent surgical tumor resection was required for diagnosis and treatment, and to avoid missing the optimal window before further decompensation.

**Fig. 2:**
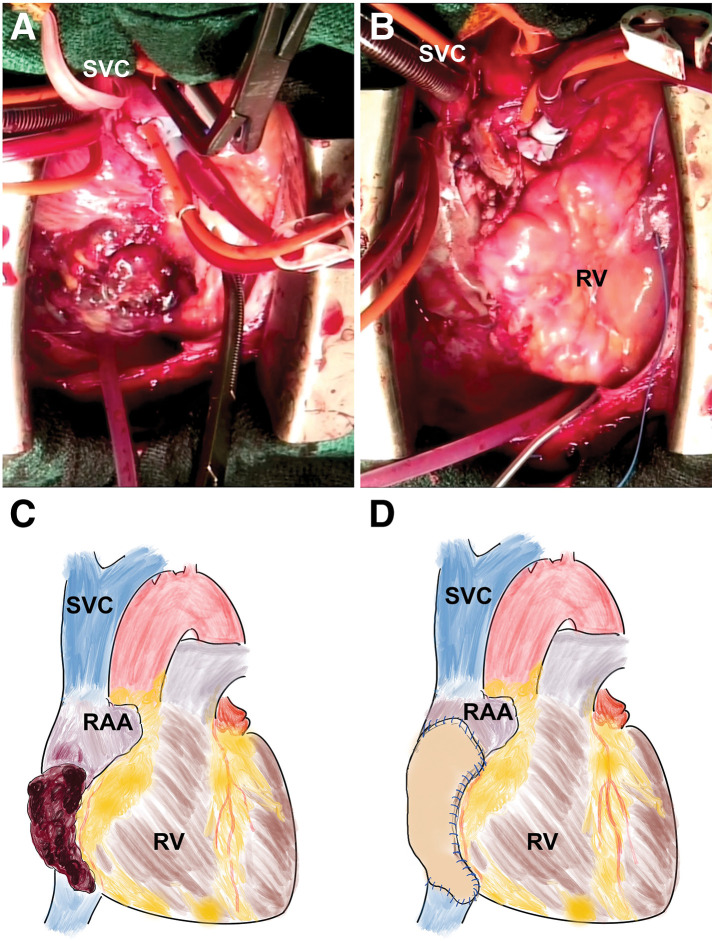
Intraoperative findings and right atrial reconstruction. (**A**) Bloody pericardial effusion and a tumor extensively infiltrating the free wall of the right atrium, with extracardiac extension visible on the epicardial surface. (**B**) Right atrial wall reconstruction using a bovine pericardial patch following en bloc tumor resection. (**C**, **D**) Schematic diagram illustrating the extent of tumor progression and the area reconstructed with a bovine pericardial patch following en bloc tumor resection. RAA, right atrial appendage; RV, right ventricle; SVC, superior vena cava

During surgery, a median sternotomy and pericardial incision revealed an irregular tumor with bloody pericardial effusion extensively involving the free wall of the right atrium, extending from near the right atrial appendage to the IVC–right atrial junction, and from the right atrioventricular groove to the interatrial groove (**Fig. 2A** and **2C**). Cardiopulmonary bypass was initiated using ascending aortic perfusion and bicaval venous drainage. The IVC was cannulated via the right femoral vein, and cardioplegic arrest was achieved successfully. The superior vena cava was snared, and the right atrium was incised at a macroscopically normal site, slightly distant from the right atrial appendage, approximately 15 mm away from the discolored lesion. The incision was extended dorsally toward the atrial septum and caudally toward the IVC. To secure an adequate operative field for achieving complete resection, the incision was initially advanced without snaring the IVC. After extending the incision in the anterior wall of the IVC superior to the pericardial reflection, the IVC was snared at the margin of the pericardial reflection to control bleeding, thereby improving visualization for the subsequent dissection while preventing air entrainment. Since the tumor had invaded the region near the right atrioventricular groove, the surrounding adipose tissue was carefully dissected using a harmonic scalpel. During this process, a branch of the right coronary artery extending toward the tumor was identified as the feeding artery and was ligated with clips. On the tricuspid valve side, the tumor margin was assessed from within the right atrial cavity, and the atrial wall was incised as far from the tumor as possible, just adjacent to the annulus, resulting in a small defect of several mm at the anterior tricuspid annulus. The tumor was resected en bloc.

Immediate intraoperative pathological examination confirmed the presence of a malignant tumor, suspected to be a leiomyosarcoma, with negative margins. The pathologist also indicated the possibility of a metastatic tumor, likely originating from a primary uterine tumor. Since the tumor was completely resected macroscopically, the anterior tricuspid annulus, right atrium, and IVC–right atrial junction were reconstructed using a bovine pericardial patch (**Fig. 2B** and **2D**). Additionally, a myocardial pacemaker lead was placed due to the potential for sinus node dysfunction.

**Fig. 3:**
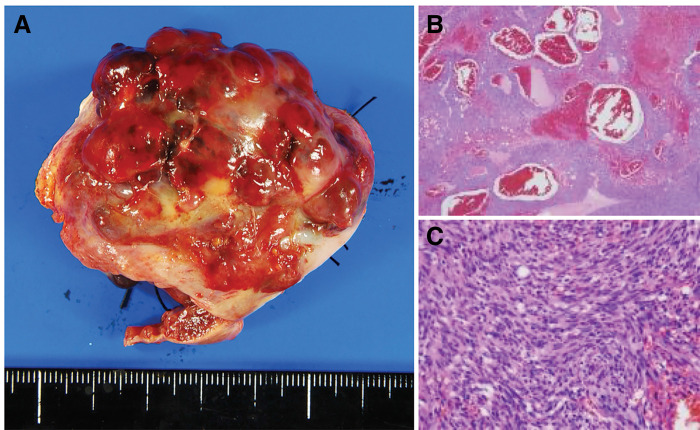
Macroscopic and histopathological characteristics of the resected tumor. (**A**) Gross specimen measuring approximately 60 mm, displaying an irregular, solid, and highly vascular appearance. (**B**, **C**) Hematoxylin–eosin staining demonstrating spindle-shaped malignant cells with marked atypia, areas of hemorrhage and necrosis, and prominent telangiectatic channels at ×40 magnification (**B**) and ×100 magnification (**C**).

Macroscopically, the tumor measured approximately 60 mm, exhibiting an irregular, solid, and vascular-rich appearance. Postoperative histological examination using hematoxylin–eosin staining revealed spindle cells with severe dysplasia invading the surrounding tissues, along with large and small telangiectasias exhibiting apparent hemorrhage and necrosis (**Fig. 3**). The mitotic count was 5–7 per high-power field, and the Ki-67 labeling index was 8%–10%. Tumor necrosis was prominent, involving approximately 70%–80% of the lesion. The surgical margin distance was 6–7 mm. Immunohistochemical testing indicated that the tumor cells were positive for CD31, CD34, and factor VIII–related antigens, confirming the diagnosis of angiosarcoma. Immunohistochemical analysis demonstrated diffuse positivity for CD31 (100% of tumor cells with strong staining intensity) and partial positivity for factor VIII (approximately 25% of tumor cells with weak staining intensity). Postoperative TTE found no residual tumors. Postoperative gynecological examination demonstrated an absence of tumors. Additionally, fluorine-18 fluorodeoxyglucose PET revealed no distant metastases or residual tumors. PET demonstrated increased metabolic activity with maximum standardized uptake values of 6.0 in the right supraclavicular region, 5.3 in the left superior mediastinum, 11.9 in the anterior mediastinum, and 4.9 in the right hilar region.

After a multidisciplinary discussion with a medical oncologist, no additional therapy was administered. Given the complete resection and potential treatment-related toxicities, close surveillance was chosen, reserving systemic chemotherapy for recurrence. Postoperative radiotherapy was not performed due to concerns about radiation-induced cardiac toxicity. All treatment options and their risks and benefits were discussed with the patient, and informed consent was obtained.

The patient was extubated on POD 1 and discharged on POD 13. She underwent pacemaker implantation for sick sinus syndrome 30 days post-surgery. Postoperative surveillance comprised CT and TTE performed every 2 months in the early phase, along with tumor marker assessment every 6 months. Three months after surgery, TTE revealed a tumor adherent to the tricuspid valve in the right ventricle, and CT demonstrated multiple lung nodules (measuring 2.5 × 2.5, 6.0 × 2.9, and 4.4 × 3.7 mm), confirming recurrence and lung metastases. Weekly paclitaxel chemotherapy was subsequently initiated.

**Fig. 4:**
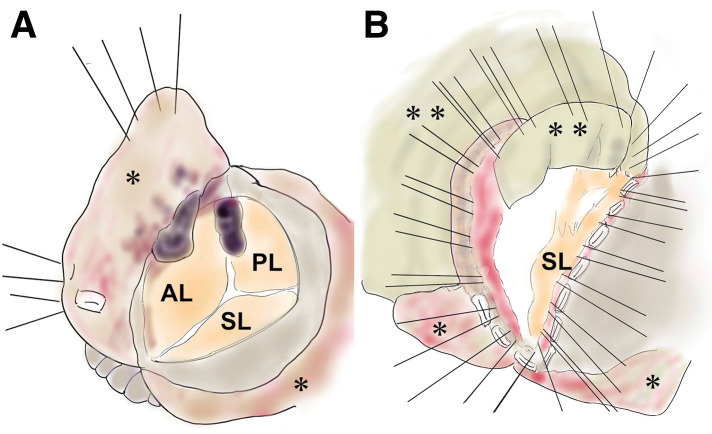
Schematic diagram illustrating tumor recurrence during reoperation and TVR. (**A**) After incision of the previously implanted bovine pericardial patch used for right atrial reconstruction, tumor involvement was identified at the annulus of the anterior leaflet and between the anterior and posterior leaflets of the tricuspid valve. (**B**) Following placement of the annular sutures, the involved portion of the previously implanted bovine pericardial patch was removed while the uninvolved portion was preserved, and the anterior leaflet and part of the posterior leaflet were resected. In the area adjacent to the tumor, myocardial resection was extended approximately 7–8 mm into the right ventricular side to secure an adequate margin. Thereafter, a new bovine pericardial patch was placed, and sutures were passed through the annulus to allow implantation of the prosthetic valve in an intra-annular position. The resected myocardial area was partially reinforced with the new bovine pericardial patch. * indicates the previous bovine pericardial patch; ** indicates the current bovine pericardial patch. AL, anterior leaflet of the tricuspid valve; PL, posterior leaflet of the tricuspid valve; SL, septal leaflet of the tricuspid valve; TVR, tricuspid valve replacement

After 2 cycles of chemotherapy, CT demonstrated marked regression of the lung nodules, which became nearly undetectable and therefore no longer measurable. However, TTE revealed apparent mobility of the tumor, indicating a high risk of embolic events, and reoperation was therefore considered. Although the risk of reoperation was substantial, the patient was relatively young with preserved physiological reserve. In addition, regression of lung metastases was observed following chemotherapy, suggesting a favorable response to systemic therapy. Based on these factors, reoperation was considered feasible, with the potential for achieving disease control. After obtaining thorough informed consent, reoperation was performed 5 months after the primary surgery. Under cardiopulmonary bypass and cardioplegic arrest, tumor resection and TVR were successfully conducted using a bioprosthetic valve (Epic, 31 mm; Abbott, St. Paul, MN, USA) (**Fig. 4**), which was selected despite the patient’s young age. The postoperative course was uneventful, and TTE revealed no residual tumors. The patient was extubated on POD 1 and discharged on POD 11. Subsequently, an additional 4 cycles of chemotherapy with weekly paclitaxel were administered. No evidence of recurrence or metastasis was identified during regular examinations until 46 months after the primary surgery, when recurrence of the right atrial tumor was detected on TTE, and liver metastasis on CT. Chemotherapy with weekly paclitaxel was reinstated, resulting in a decrease in the number of recurrent and metastatic lesions.

Progressive liver and lung metastases were observed 62 months following primary surgery, prompting a change in the chemotherapy regimen to eribulin. Additionally, 65 months after the primary surgery, the patient sought a second opinion from another physician and subsequently received radiation therapy and chemotherapy with pazopanib instead of eribulin. Unfortunately, she experienced intra-abdominal bleeding complicated by repeated disseminated intravascular coagulation and succumbed to multiple organ failure 68 months after the primary surgery.

## DISCUSSION

PCA is a rare and highly aggressive malignant tumor,^5,6)^ whose most common clinical manifestations include dyspnea (50%–80%), pericardial effusion (29%–56%), and chest pain (10%–39%).^5)^ Tumors typically arise in the right atrium in 70%–100% of cases, with a mean size ranging from 5.8–7.2 cm.^5,6)^ Furthermore, metastasis is common at the time of diagnosis, particularly in the lungs (20%–55.6%), liver (10%–22.2%), and bone (10%–20%).^5)^ The prognosis remains poor, with a median survival time of 7.22 months and a 5-year survival rate of 10.2%.^7)^ Treatment typically involves surgical resection when feasible, often in combination with chemotherapy and/or radiotherapy; however, complete resection is rarely achieved.^1,6)^

Complete resection is the most critical factor for attaining improved long-term outcomes, as angiosarcoma is largely unresponsive to existing chemotherapy and radiation therapy. However, complete resection is often challenging due to extensive involvement of vital cardiac structures, including the atria, ventricles, tricuspid valves, and coronary arteries.^8,9)^ Successful complete resection typically necessitates reconstruction using bovine pericardial patches or autologous pericardium.^10)^ Notably, research has shown that patients who undergo complete resection have significantly better survival rates compared to those who undergo incomplete resection, with median overall survival rates of 17 and 5 months, respectively.^1)^

The primary tumor location in cardiac angiosarcoma is not consistently deemed an independent prognostic factor; however, it strongly influences the feasibility of achieving complete resection. Right atrial tumors, which represent most cases, frequently exhibit extensive local invasion at diagnosis, limiting the likelihood of negative-margin resection. Given that complete resection has been consistently demonstrated as the most significant determinant of survival, the primary tumor location may indirectly affect prognosis via its impact on resectability.

Nonetheless, recurrence is common even after complete resection. Although most available data are derived from studies of primary cardiac sarcomas rather than angiosarcomas alone, several studies have demonstrated that re-resection for recurrent disease is feasible and can improve outcomes.^11–13)^ In those studies, patients who underwent aggressive management of recurrence, including surgical resection, radiofrequency ablation, or radiotherapy, exhibited a longer median survival (47 months) compared with those who received no further intervention (25 months).^13)^ Combined approaches using re-resection with targeted therapy, such as pazopanib, have shown promise in achieving prolonged survival and complete remission.^14)^ Overall, aggressive multimodal treatment, including re-resection, optimizes outcomes despite the challenging prognosis.

In the present case, tumor invasion of the tricuspid annulus was suspected during the initial surgery. Therefore, tumor resection was performed as close to the annulus as possible, followed by reconstruction using a bovine pericardial patch. However, recurrence subsequently occurred, necessitating repeat surgery with tumor resection and TVR; therefore, upfront TVR may be a reasonable option in cases with a high risk of local recurrence near the tricuspid valve, particularly from the perspective of securing an adequate safety margin. In our case, to guide intraoperative decision-making, the right atrial tissue adjacent to the tricuspid annulus was submitted for pathological evaluation, including intraoperative frozen section analysis, which confirmed negative surgical margins. In addition, considering the potential risk of TVR-associated worsening of right ventricular function, we decided to preserve the native tricuspid valve at the initial operation.

Systemic and locoregional therapies for cardiac angiosarcoma present significant challenges due to the tumor’s rarity and aggressive biology, resulting in the lack of a standardized treatment regimen. Various chemotherapy protocols have been utilized, including doxorubicin, dacarbazine,^15)^ liposomal doxorubicin,^2)^ paclitaxel,^16)^ and docetaxel.^17)^ Additionally, radiation therapy encompasses conventional radiotherapy and stereotactic body radiation therapy.^16)^ Moreover, innovative techniques, such as PET/MRI-guided adaptive radiotherapy, have demonstrated potential for achieving substantial tumor volume reduction when combined with paclitaxel.^18)^ Recent studies on cardiac angiosarcoma reveal limited specific targeted therapy options for this rare and aggressive malignancy. While targeted therapies directed at angiogenic mechanisms and molecular abnormalities show promise for improving outcomes,^19)^ most research focuses on broader sarcoma treatments rather than cardiac-specific approaches. Anti-angiogenesis therapy has demonstrated favorable efficacy in sarcomas generally, and inhibitors targeting various pathways, including cyclin-dependent kinase 4 and 6, poly(ADP-ribose) polymerase, insulin-like growth factor 1 receptor, and mechanistic target of rapamycin, are under clinical evaluation.^20)^

Paclitaxel was selected as the initial systemic therapy in this case. Although doxorubicin-based regimens are commonly used for soft tissue sarcomas, its clinical utility is limited by dose-dependent cardiotoxicity.^21)^ In contrast, paclitaxel has demonstrated efficacy in angiosarcoma, which is recommended in the NCCN Clinical Practice Guidelines in Oncology for Soft Tissue Sarcoma (Version 1.2026).^22)^ Given its favorable tolerability profile and feasibility for continued administration, paclitaxel was considered an appropriate first-line agent in this patient. Subsequent transitions to eribulin and pazopanib were made in response to disease progression during treatment. In addition, treatment selection at each stage considered tolerability and the feasibility of continued administration, reflecting a sequential treatment strategy commonly employed in advanced sarcoma management.

In this case, no adjuvant therapy was administered immediately after the initial surgery. First, there is no clear evidence demonstrating the survival benefit of postoperative adjuvant chemotherapy after complete resection of soft tissue sarcomas including angiosarcomas. Considering that complete resection had been achieved and the potential for treatment-related toxicities, a close surveillance strategy was adopted, reserving systemic chemotherapy for recurrence. However, even in cases with complete resection, a high rate of recurrence has been reported, and several studies have described long-term survival following multimodal treatment including early postoperative chemotherapy. Therefore, although no definitive guidelines exist, early adjuvant chemotherapy may be a potential treatment option. However, treatment-related toxicities remain a significant concern, particularly in the early postoperative period. Thus, the indication for adjuvant therapy should be determined on a case-by-case basis, considering the patient’s general condition, cardiac function, and wound healing status. Furthermore, treatment decisions should be centered on a shared decision-making process with the patient under adequate informed consent.

Several studies have documented the long-term survival of PCA following complete or repeat resection, combined with multimodal therapy.^13,23,24)^ Despite the overall poor prognosis associated with angiosarcoma, these reports consistently emphasize that complete resection and effective local or systemic control of limited recurrent or metastatic disease are critical for prolonging survival. Furthermore, most long-term survivors are relatively young and exhibit sufficient physiological reserves to tolerate aggressive multimodal therapies. **Table 1** depicts a comparison with previously reported long-term survivors. In contrast to these cases, the present case demonstrates several unique features. Despite originating in the right atrium—where tumors are typically associated with extensive local invasion and limited resectability—complete resection was achieved successfully. Furthermore, no adjuvant chemotherapy was administered after initial surgery; however, relatively long-term survival was obtained through re-resection and subsequent systemic chemotherapy following recurrence. These findings suggest that a staged multimodal strategy may contribute to improved outcomes even in patients with right atrial angiosarcoma.

**Table 1:** Clinical characteristics and outcomes of long-term survivors with cardiac angiosarcoma, including the present case

Reference	Primary tumor location	Metastasis at diagnosis	Complete resection (R0)	Concomitant procedures	Neoadjuvant chemotherapy	Adjuvant chemotherapy	Radiotherapy	Recurrence	Subsequent treatments	Outcome	Survival duration (month)
Centella et al. (2005)^23)^	LA	None	Yes	LA wall resection	No	Ifosfamide, adriamycin, methotrexate	No	LA (24 months, reported as 2 years)	Re-resection + chemotherapy	Dead	48 (reported as 4 years)
Bellitti et al. (2013)^24)^	RA	None	Yes	RA wall resection + reconstruction (autologous pericardium)	No	Gemcitabine, docetaxel	Yes	Lung (suspected, 9 months)	Chemotherapy + targeted therapy	Alive with disease	At least 27
Bakaeen et al. (2009), Case 1^13)^	RA	Lung	No (R1)	RA wall resection + reconstruction (bovine pericardium), bilobectomy 1 month later	AI	Paclitaxel, doxorubicin, DTIC, gemcitabine	Yes	IVC (5 months), brain (39 months)	Re-resection (R2 at liver), resection of brain metastasis	Dead	58
Present case	RA	None	Yes	RA wall resection + reconstruction (bovine pericardium)	No	No	No	Lung (3 months)	Re-resection + chemotherapy	Dead	68

DTIC conventionally refers to dacarbazine; however, in the original study by Bakaeen et al.,^13)^ the abbreviation was used to indicate a combination regimen comprising doxycycline, Taxol (paclitaxel), ifosfamide, and cyclophosphamide.

AI, adriamycin (doxorubicin), ifosfamide; IVC, inferior vena cava; LA, left atrial; RA, right atrial

Several factors likely contributed to the extended survival period of 68 months, which can be categorized into tumor-, treatment-, and patient-related factors. Tumor-related factors include the achievement of negative surgical margins following repeat resection and the tumor’s responsiveness to systemic chemotherapy. Preoperative chemotherapy resulted in partial regression of pulmonary metastases, suggesting a degree of chemosensitivity and effective systemic disease control at the time of reoperation. Although the prognostic significance of the histologic grade in cardiac angiosarcoma remains unclear, the relatively low-to-moderate proliferative activity observed in this case may have contributed to its clinical course. Treatment-related factors also played a crucial role. Although the initial indication for reoperation was based on the risk of embolic events, the proactive decision to proceed with repeat surgery enabled complete resection of the recurrent tumor. Moreover, continuous postoperative adjuvant chemotherapy may have contributed to sustained disease control, maintaining remission for 41 months following the second surgery. Patient-related factors, particularly the relatively young age and preserved physiological reserve, likely allowed tolerance of aggressive multimodal treatment, including repeat cardiac surgery and prolonged systemic therapy. Collectively, the combination of complete surgical resection, favorable response to chemotherapy, and adequate patient resilience may have acted synergistically to achieve durable disease control and unusually prolonged survival in this highly aggressive malignancy.

## CONCLUSIONS

This case illustrates that, even in PCA, which typically has a dismal prognosis, long-term survival may be achieved through an individualized, aggressive multimodal approach. Complete surgical resection was the cornerstone of treatment. Re-resection for localized recurrence should be considered when technically feasible and the patient’s overall condition supports aggressive intervention. In this instance, preoperative chemotherapy resulted in regression of pulmonary metastases, followed by curative resection and ongoing adjuvant therapy. These treatments likely acted synergistically to maintain disease control, culminating in an exceptional 68-month survival. These findings underscore the potential advantages of proactive surgical intervention and sustained systemic therapy in select patients with otherwise fatal malignancies.
